# Long Island Veterinary Society

**Published:** 1891-01

**Authors:** D. S. Breslin

**Affiliations:** Secretary


*Long Island Veterinary Society.*—A regular meeting of the Long Island Veterinary Society was held on Wednesday evening, December 17th, 1890, at 8 o'clock, at No. 74 Adams Street, the President, Dr. George H. Berns, in the chair.

The following members were present: Drs. George H. Berns, George F. Bowers, J. F. Mustoe, Philip Newman, Thomas M. Buckley, Samuel Atchison, D. S. Breslin, William H. Pendry, Roscoe R. Bell, George G. Vanderveer, Charles Jamieson.

The minutes of the previous meeting were read and approved.

The Treasurer, Dr. George F. Bowers, read his report for the term ending January 1st, 1891, the report showing a balance of cash on hand of $44.83. The report was received and adopted. The Board of Censors and the Board of Trustees had no report to make.

Dr. William H. Pendry was instructed to file the yearly report of the society's condition in the county clerk's office, thus complying with the law on the subject.

The next order of business being reading of papers, Dr. William H. Pendry read an interesting paper entitled "Uniform Standard of Veterinary Education," as follows:

UNIFORM STANDARD OF VETERINARY EDUCATION.

In selecting a subject for your consideration this evening I may possibly disappoint some of those who have a more practical turn of mind; yet the question submitted I consider one of the most vital to the profession, and it deserves your full and most serious attention. Some few years ago I tried, in a rough way, to bring about a discussion on veterinary education, but very imperfectly succeeded, for what reason I do not know; perhaps it was that comments might have added a further blur to the poor opinion that many have of some graduates, whose very diversity of knowledge lends color to the impression that the common educational ability has a remarkably wide range. Why this condition of affairs? Why this silent taunt at so noble and so scientific a profession whose field of usefulness and work has such an immense range, is hard to conceive. Doubtless the vast difference between an ordinary veterinary surgeon and an educated veterinarian is as yet beyond the comprehension of the general public. As a rule, the one's usefulness is limited to his particular line, the other knows no bounds in the field of his profession, whose extensiveness lies beyond the imagination of an ordinary mind, and can only be conceived by an ideal realization or a scientifically educated person. How often do those who are loyal to their profession become disgruntled with their own knowledge and absolutely disgusted with that displayed by others! Do not imagine that I here intend to start out on a crusade against the different schools of this or any other country, in contending that this one or that one has turned loose those who have helped to germinate such an idea as the foregoing. Yet I cannot refrain from saying, generally, that therein *may* lie some of the cause; still, I consider there is a more direct cause in that I believe that the existence of so many veterinary schools—and how many have sprung up within the last ten or fifteen years, either as an appendage to other institutions, or even veterinary colleges dependent upon their speculative results—has created a dangerous competition. And so much has this been the case that in many instances there has sprung up such rivalry that that very spirit, if left alone to run its own natural course, which lies so close to the crater that the slightest volcanic eruption might bring down such a shower of public contempt that the profession would be buried more deeply than Pompeii. As I have stated, I do not wish to assail any particular school, but simply desire to deal with the question in a general way; yet it cannot be denied where one offers to produce graduates for a less fee and in less time, the latter being often of greater moment to the interested party than the actual cash paid down. There must be some difference in that which is tendered for a consideration, not but what I am perfectly willing to admit that there are exceptions even to this rule; as some seem more adapted for their calling, coupled with a desire to increase their knowledge at every opportunity. But this, I claim, is the exception, and I am sure that a large majority of young graduates will answer—perhaps in silence—a conscious inquiry, is his knowledge consistent with a truly educated veterinarian, in the negative. Yet so many are satisfied if after leaving school they can, under the shelter of a diploma, treat a punctured wound of the foot, a case of colic or even diagnose a case of lameness; and this ends their ambition. Are such men true representatives of a profession that can be made one of the most scientific under the sun? Surely there must be times when such men feel within themselves and realize their inefficiency. The writer is free to admit that many times he has exclaimed to himself that he did not know a thing; the last word being preceded by an emphasis that was somewhat unparliamentary. Yet he is a reader and more or less studiously inclined. This is said with no reflection on his Alma Mater, for whom he has the most profound regard; but I do say without any hesitancy that the course, as generally given in the colleges of the present day, is not on a par with the importance of the profession. This is bad enough, but even this wrong is lost in significance when compared with a greater one—the desire to turn out large classes. When I speak so reflectingly I am not unmindful of the fact that in no country has the veterinary profession made greater strides than in the United States during the last twenty-five years; yet our success must not submerge our judgment when certain reformation is called for by reason of circumstances that even might be attributed to such advancement, and which is born of a desire to too quickly show results in too short a time. I realize that these views may be considered selfish by those who have limited means and time, who doubtless are making great sacrifices to join our profession, with the determination to do it honor. Such a condition of affairs, brought about by such circumstances, finds no keener sympathizer than the writer, and it is to such men that I would do honor by protecting the profession they have chosen by raising them and it to a higher plane. How is this to be done? My answer is, by having a uniform standard of education. And how shall that be accomplished? For a solution of that problem let me refer you to the proceedings of the United States Veterinary Medical Association recently held in Chicago. Not that the idea of a National Board of Veterinary Examiners was conceived there, it having been written upon and talked about years ago, but that it received its first serious consideration at the hands of a large representative body of veterinarians; and it is seriously hoped that the fruits of such conception will quickly come to light and produce a healthier state of affairs. Now comes the difficult part of the problem; what authority shall create that Board and how great shall be its power? If the plan were only to be carried out in one State, it would be much easier; but it would avail nothing if the movement was not a National one. The idea of having examiners appointed by the Department of Agriculture in Washington is undoubtedly a good one, yet I have no doubt that some will be afraid of politics creeping in; but safeguards could be easily thrown around such a Board by adopting the same plan that is employed in the formation of the Board of Elections. So far so good; but how about the conflict of State authority? Certain colleges in certain States have been invested by charter or otherwise with authority to grant diplomas and to create a National Board of Examiners, who alone shall have that authority. This would conflict with State laws. Yet, if they cannot be deprived of this power, I see no reason why a National Board of Examiners cannot be created to be known as the Veterinary College of America, whose function alone shall be to grant diplomas to such graduates of the different colleges who shall pass a post examination before them, the value of which would be so established that all others would soon become professionally obliviated.

No time should be lost in having a bill introduced in Congress creating such a body and investing them with authority to hold examinations twice a year—say one in Chicago for Western schools, and one in Washington for those in the East. I think such a bill would receive little or no opposition, provided it did not directly deprive schools of the graduating power already invested in them. The value of the profession to the nation has too recently been demonstrated not to receive the support of the Government when it can be positively asserted that the success or non-successes of the cotton industries of this vast country depend very largely upon the proficiency of its veterinarians, saying nothing about the relationship of the profession to public health in regard to the consumption of meat and milk.

I have not had the time to give this subject that it so justly claims; but, short as this paper is, and crude as the ideas and comments are, I trust they will be the means of starting a discussion that may lead to something tangible.

An interesting discussion followed, participated in by Drs. R. R. Bell, George H. Berns, J. F. Mustoe, George F. Bowers, Thomas M. Buckley and William H. Pendry, after which a hearty vote of thanks was tendered to the essayist.

The election of officers being next in order, the following gentlemen were elected to the various offices for the following year: President, Dr. R. R. Bell; First Vice President, Dr. Samuel Atchison; Treasurer, Dr. George F. Bowers; Secretary, Dr. D. S. Breslin; Board of Censors, Drs. George H. Berns, Philip Newman, H. Housman, Thomas M. Buckley, J. F. Mustoe.

Bills for type-writing, stationery and postage, amounting to $4.10, were ordered paid. A vote of thanks was unanimously tendered the retiring officers, particularly the President, Dr. George H. Berns, for their efficient services in behalf of the society.

The chair appointed as essayist for January meeting Dr. George V. Vanderveer.

The meeting then adjourned.

D. S. BRESLIN, D.V.S.,
*Secretary.*

